# Portomesenteric Vein Thrombosis after Bariatric Surgery: An Online Survey

**DOI:** 10.3390/jcm10174024

**Published:** 2021-09-06

**Authors:** Francesco Maria Carrano, Sylvia Weiner, Moustafa Elshafei, Saleem Ahmed, Toghrul Talishinskiy, Valeria Tognoni, Kamal Mahawar, Nicola Di Lorenzo

**Affiliations:** 1 PhD Program in Applied Medical-Surgical Sciences, University of Rome “Tor Vergata”, 00133 Rome, Italy; carranofm@gmail.com; 2 Department of Surgical Sciences, University of Rome “Tor Vergata”, 00133 Rome, Italy; vtognoni@gmail.com; 3 Department of Metabolic Surgery, Krankenhaus Nordwest, Steinbacher Hohl 2-26, 60488 Frankfurt, Germany; sylvia.weiner@gmx.de (S.W.); elshafei.moustafa@gmail.com (M.E.); 4 Department of Upper Gastrointestinal and Bariatric and Metabolic Surgery, Tan Tock Seng Hospital, Singapore 308433, Singapore; medicsaleem@gmail.com; 5 Hackensack University Medical Center, Hackensack, NJ 07601, USA; talishinskiy.toghrul@gmail.com; 6 Department of Surgery, Sunderland Royal Hospital, Sunderland SR4 7TP, UK; kmahawar@gmail.com

**Keywords:** portomesenteric vein thrombosis, bariatric surgery, portal vein thrombosis, sleeve gastrectomy, RYGB

## Abstract

Portomesenteric vein thrombosis (PMVT) is a rare post-operative complication of bariatric procedures, occurring in between 0.3% and 1% of cases. A structured questionnaire consisting of 27 items was available online to members of the International Federation for the Surgery of Obesity and Metabolic Disorders (IFSO) to investigate the occurrence of PMVT. A total of 89 bariatric surgeons from 61 countries participated. Twenty-six (29.21%) reported at least one case of PMVT (46.15% males; 53.84% females). The surgery most associated with PMVT occurrence was sleeve gastrectomy (84.6%), followed by Roux-en-Y gastric bypass (RYGB) (7.69%), and laparoscopic adjustable gastric banding (LAGB) (7.69%). The time gap between surgery and PMVT was 19.28 ± 8.72 days. The predominant symptom was abdominal pain in 96.15% of patients, followed by fever in 26.9%. Complete occlusion of the portal vein was reported in 34.6% of cases, with involvement of the portal system in 69%, extension to the superior mesenteric district in 23%, and extension to the splenic vein in two patients (7.7%). Our survey, which is the largest regarding PMVT to date, revealed a diffuse lack of standardization in the choice, duration, and dosing of prophylaxis regimens as well as treatment modalities, reflecting the literature gap on the topic.

## 1. Introduction

Bariatric surgery (BS) is recognised as the most effective treatment for morbid obesity [1] and its associated co-morbidities [2]. The global volume of bariatric procedures has increased considerably over the last decade, reaching approximately 833,000 operations in 2019, of which 391,423 (47.0%) were sleeve gastrectomy procedures (LSG), 294,530 (35.3%) Roux-en-Y gastric bypass (RYGB) operations, 70,085 (8.4%) gastric banding procedures, 30,914 (3.7%) one-anastomosis gastric bypass procedures (OAGB), and 2744 more invasive biliopancreatic diversions/sleeve gastrectomies with duodenal switch (BPD/DS) that represent less than 1% of bariatric procedures [3]. Moreover, the field of bariatric surgery is constantly evolving towards less invasive endoscopic approaches [4].

Portomesenteric venous thrombosis (PMVT) refers to a partial or complete obstruction of the portal venous system in the intra- or extra-hepatic venous tract or affecting the splenic or superior mesenteric veins. Its consequences can be severe, and include ascites (62%), esophageal varices (58%), terminal gastroesophageal bleeding (47%), bowel infarction, and death [5]. The most common reported causes of PMVT are myeloproliferative disorders, deficiencies of anticoagulant proteins, prothrombotic gene mutations, cirrhosis with portal hypertension, and hepatocarcinoma [6].

Though many cases of PMVT have been reported in the scientific literature following BS, its presentation, pathogenesis, diagnosis, and management remain unclear [7]. The aim of this survey was to understand the incidence, presenting symptoms, possible risk factors, diagnosis, treatment, and outcomes of PMVT.

## 2. Materials and Methods

A study group consisting of an international panel of surgeons with experience in bariatric surgery drafted a structured questionnaire that consisted of 27 items (Online Appendix) divided into the following sections: (I) Participant experience; (II) Patient characteristics; (III) Coagulation state; (IV) Pharmacological anamnesis; (V) Surgical procedure; (VI) Pre-operative data; (VII) Intra-operative data; (VIII) Post-operative data; (IX) Portal vein thrombosis diagnosis; (X) Imaging studies; (XI) Thrombosis site; (XII) PMVT treatment; and (XIII) Outcome. The survey questions were close-ended. Consent of survey participants was obtained with a direct question in the survey. If participants had at least one case of PVT in their experience, they were required to respond to all questions for their responses to be registered, otherwise the survey was terminated. Patients were identified using surgeons’ personal records.

### 2.1. Sampling Plan and Invitation

Members of the European Chapter of the International Federation for the Surgery of Obesity (IFSO-EC) were invited to participate in the survey through: (I) Email invitation to IFSO-EC members and affiliated individuals; or (II) The IFSO-EC email newsletter sent to IFSO members and affiliated individuals. Individual invitations were sent. Participants were informed that data was anonymised, confidential, and were provided a web link directing to the survey instrument. For the survey we used Google Forms, and it was hosted by Google LLC (Mountain View, CA, USA).

### 2.2. Statistical Analysis

A recorded summary of the results for each question was generated. Standard descriptive statistics were used.

## 3. Results

A total of 89 bariatric surgeons from 61 countries participated. Survey response rate was 4%. Most participants were based in Europe (91%); 65.16% (58) were practicing in high-volume bariatric centres (>100 bariatric operations per year), while the remaining 34.84% (31) worked in hospitals with a medium- or low-volume of bariatric procedures.

Twenty-six (29.21%) reported at least one case of PMVT in their clinical practice after bariatric surgery. Of those, only five (19.23%) worked in low- or medium-volume institutions, while twenty-one surgeons (80.76%) worked in high-volume bariatric centres.

The cases of PMVT submitted occurred in twelve males (46.15%) and fourteen females (53.84%). The median pre-operative body mass index (BMI) was 44.55 ± 5.32 kg/m^2^; the median pre-op weight was 129.26 ± 20.79 kg (Table 1). Among the patients that received pre-operative study for altered coagulation states, three had a known coagulation disorder (12%), two of them had protein S deficiency, while one was reported as a nonspecific prothrombotic state. Three patients had a previous history of deep vein thrombosis and one of these had protein S deficiency. Four patients were under medication with selective serotonin reuptake inhibitors (SSRIs) (16.6%).

**Figure 1:**
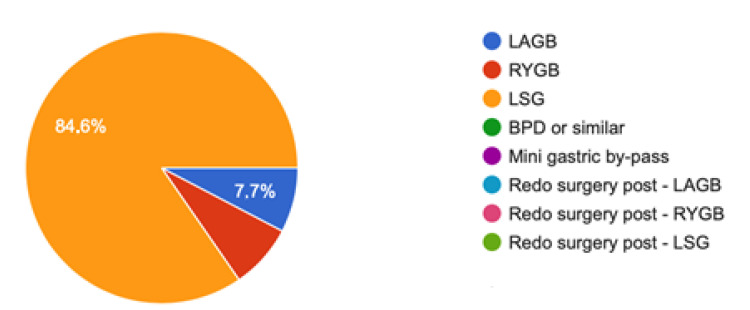
Procedures associated with PMVT. PMVT: Portomesenteric vein thrombosis; RYGB: Roux-en-Y gastric bypass; LSG: sleeve gastrectomy procedures; BPD: biliopancreatic diversions LAGB: Laparoscopic Adjustable Gastric Banding.

The surgery most associated with PMVT occurrence in this survey was sleeve gastrectomy, with twenty-two out of twenty-six cases (84.6%); two patients developed PMVT after RYGB (7.69%) and two after LAGB (7.69%), as shown in Figure 1.

The median operative time reported was 64.76 ± 27.6 min with no intraoperative complications.

The time gap between surgery and PMVT was 19.28 ± 8.72 days. Antithrombotic prophylaxis was administered to all these patients with low molecular weight heparin (LMWH) and/or compression stockings.

The predominant symptom (Table 2) was abdominal pain, seen in twenty-five out of twenty-six patients (96.15%), followed by nausea and vomiting that were reported in twelve and seven cases respectively (46.15% vs. 26.9%). Fever was present in seven patients (26.9%). Five patients showed anorexia (19.2%). Diarrhoea was present in three patients (11.5%) and, in one case, intestinal bleeding (3.8%) was the initial presentation.

**Figure 2:**
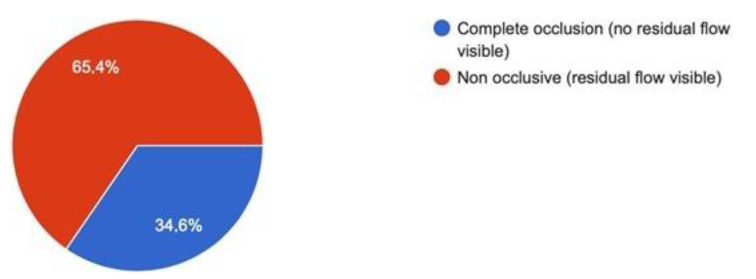
Grade of occlusion of the portal system.

**Figure 3:**
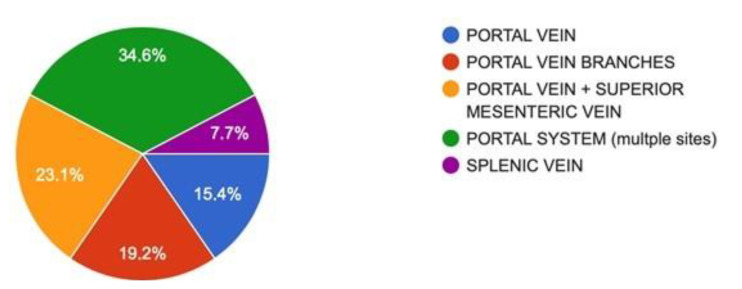
Extent of portal system involvement.

Diagnosis was achieved after a contrast-enhanced abdominal CT scan in all cases, alone or in combination with abdominal doppler ultrasound. In 34.6% of the patients, there was complete occlusion of the portal system (Figure 2). Figure 3 provides percentages for the extent of portal venous system involvement.

All patients received medical treatment with one of the following: unfractionated heparin, LMWH or oral anticoagulant. Endovascular thrombolysis was performed in one patient (3.8%). Two patients required a diagnostic laparoscopy (7.7%), but no bowel resection was required, and no deaths occurred. All patients were treated successfully and achieved complete resolution of the thrombosis.

## 4. Discussion

Portomesenteric vein thrombosis is a known complication after bariatric surgery. In our survey, only 29.21% of responders reported at least one case of PMVT in their practice, most of them coming from high-volume bariatric centres, with no significant differences in gender. Although rare, its incidence has been increasing on par with the number of bariatric procedures performed worldwide, occurring in 0.3–0.4% of cases, mostly after LSG (78.9%) and RYGB (13.8%) [7,8]. Our survey reflects the data in the literature, with LSG being the procedure most associated with PMVT, followed by RYGB and LAGB.

**Figure 4:**
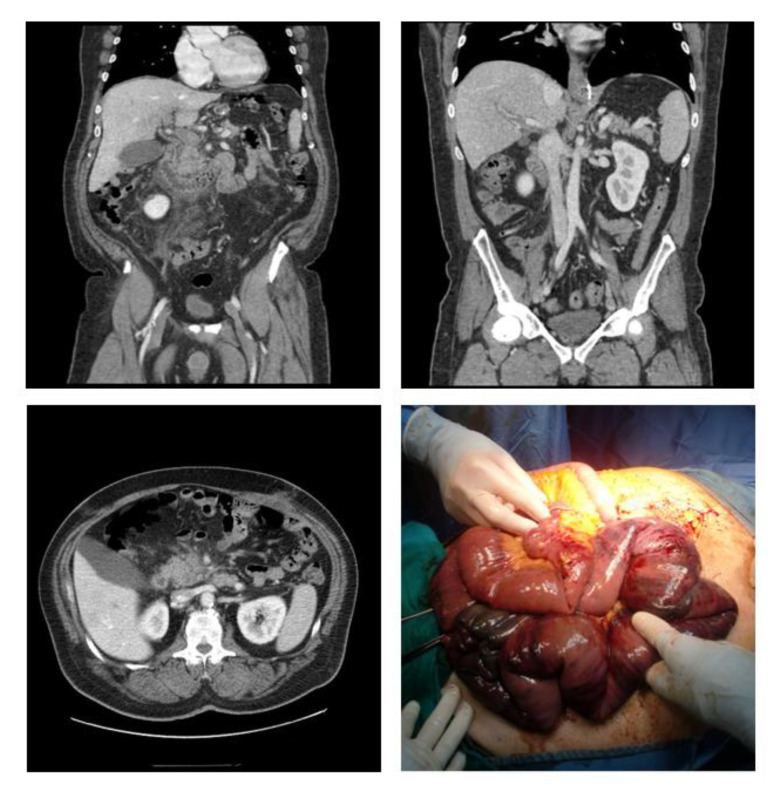
Case of portal vein thrombosis after biliopancreatic diversion; CT scan showing extended PVT associated with bowel ischemia, and related intraoperative findings. PVT: Portal Vein Thrombosis.

PMVT usually begins with a mild presentation that can be managed conservatively. However, in severe cases, it can potentially be lethal due to the risk of associated bowel ischemia (Figure 4) and potential late risk of chronic bilioportal cholangiopathy that can lead to small bowel resection and even liver transplantation [9]. Moreover, the occurrence of PMVT is among the factors that influence early postoperative liver function capacity of patients after bariatric surgery [10].

In our survey, the most common presentation was abdominal pain in over 96% of cases, followed by nausea in almost half of cases, and vomiting in 27%. Less than 30% of patients had a fever. Less frequent initial presentations included anorexia, diarrhoea, and intestinal bleeding. Similar findings are reported in a systematic review by Shoar S. et al., in which the most common presentations of PMVT were abdominal pain (82.7%), nausea/vomiting (38.2%), leukocytosis (20%), and fever (12.7%). Other less common presenting symptoms were tachycardia (10.9%), increased liver function tests (11.8%), elevated erythrocyte sedimentation rate/C-reactive protein level (10%), and malabsorption (9.1%).

Although the only predominant symptom in our series was abdominal pain, the early recognition of PMVT with the use of abdominal ultrasound and contrast-enhanced CT scans was important to provide early conservative treatment. All patients received medical treatment with either unfractionated heparin, LMWH, or oral anticoagulant. Only one patient required endovascular thrombolysis, while two underwent a diagnostic laparoscopy without the need for any bowel resection. Similar treatment strategies are reported in the literature, with interventional radiology techniques allowing relatively easy treatment of portal thrombosis in non-cirrhotic patients [5,11].

No clear predisposing factor or causative agent emerged from our survey. Among the patients that received specific pre-operative coagulation study, 12% had a known coagulation alteration, and 11.5% had a previous history of deep vein thrombosis. The use of SSRIs was reported in 16.6% of patients with PMVT, although no conclusions can be drawn on any causative relationship between SSRIs and PMVT due to the low number of cases. Several causes have been suggested in the past, although none have been identified as solely responsible. A status of hypercoagulopathy is retrieved in less than half of patients, with several different coagulation factors implicated in its genesis; thus, the possible cause remains unknown in the majority of cases. Recently, a case of PMVT after RYGB has been reported in a young patient with a SARS-CoV-2 viral infection [12], which is known to lead to a hypercoagulopathic state that can result in PMVT [13] and mesenteric ischemia [14]. Previously, other viral infections have been reported in PMVT, including Epstein–Barr and cytomegalovirus, as well as in healthy and immunocompetent patients [15]. The role of viral infections in the genesis of PMVT after bariatric surgery has not been investigated yet. Several different mechanisms have been held responsible for cases of PMVT occurring after LSG, including rearrangement of the splanchnic venous flow secondary to ligation of the short gastric vessels [16], oedema of the sleeve causing venous obstruction or dehydration, or compression of venous flow by liver retraction [8]. Although the reduction of portal venous flow is a known factor for the occurrence of PMVT [17], it is yet to be clarified if transitory flow reduction during surgery can be sufficient to initiate PMVT. A recent study by Osman et al. associated LSG with greater reduction in portal venous peak systolic flow velocity in the early postoperative period, compared to other bariatric procedures, although no PMVT occurred in the study cohort [18]. Induction of a pneumoperitoneum with an intra-abdominal pressure of more than 14 mmHg has been demonstrated to reduce portal venous blood flow by 50%, that is further reduced by the prolonged reverse-Trendelenburg position. A hypercapnic state may also cause mesenteric vasospasm that eventually reduces venous blood flow, increasing the risk of blood clot formation. However, these factors exist for many other laparoscopic procedures that are not associated with an increased risk of PMVT [19] and can hardly explain the mean occurrence of PMVT 19 days after surgery. Some authors also hypothesise a role of high energy ultrasonic devices that may favor thrombus formation and dissemination through the portal venous system due to the mechanical and thermal energy transmitted to blood vessels and surrounding tissues [20,21], although there is no strong evidence currently supporting this theory, as no dedicated studies have investigated this particular aspect and PMVT cases are reported when only bipolar energy devices were used. In our survey, no particular risk factor was predominantly associated with PMVT.

We believe that the analysis of the data collected with the survey can offer a general and more realistic overview of the occurrence of PMVT in the bariatric population compared with studies available in the literature—most of them retrospective and on small cohorts of patients. An interesting finding of our study is that the time between surgery to PMVT presentation was 19.28 ± 8.72 days. This data suggests that a short course antithrombotic prophylaxis of less than 15 days may not be sufficient to lower the risks of such a complication. Moreover, this is consistent with the experience of Manish et al. on the systematic use of extended chemoprophylaxis, that decreased the rate of PMVT from 0.3% to 0.1% without a significant increase in the number of bleeding episodes [8]. A clear fact emerging from this survey is the great heterogeneity not just in choice but also in the duration and dosing of antithrombotic prophylaxis. It is also worth considering that currently available calculators used to predict the risk of VTE do not take into consideration PMVT, reflecting a further lack of tools to standardise the approach on the prevention of PMVT and VTE in general.

One limitation of our study is the low response rate to the questionnaire. We think that this might be due to the rarity of the condition so that most surgeons, and even bariatric surgeons, would not have encountered it in their practice. The diverse results collected by our survey are also representative of the lack of quality evidence regarding PMVT, as most of the studies have different inclusion criteria, do not follow a standardised surgical technique (rendering comparison difficult), adopt different types of thrombophylactic regimens, as well as different diagnostic and treatment algorithms. For this reason, prospective studies with sufficient power should be encouraged to fill this important gap in the literature.

## 5. Conclusions

PMVT is a rare complication in bariatric surgery, however, it can have severe consequences. Early detection through cross-sectional imaging is fundamental for positive treatment results. Our survey that represents the largest survey on PMVT to date, revealed a diffuse lack of standardization in the choice, duration, and dosing of prophylaxis regimens as well as treatment modalities, reflecting the literature gap on the topic. Further studies should be encouraged to address this important issue.

## Figures and Tables

**Table 1:** Survey patient characteristics.

*N* Patients	26
Surgery	LSG 22/RYGB 2/LAGB 2
Sex	14 F/12 M
Median pre-op weight	129.26 ± 20.79 kg
Median BMI	44.55 ± 5.32 kg/m2
Coagulation disorder	3 (12%)
Previous DVT	3 (12%)
SSRIs	4 (16.6%)

BMI: Body Mass Index; DVT: Deep Vein Thrombosis; LAGB: Laparoscopic Adjustable Gastric Banding; LSG: Laparoscopic Sleeve Gastrectomy procedures; RYGB: Roux-en-Y Gastric Bypass.

**Table 2:** PMVT presenting symptoms.

Symptom	Patients (%)
Abdominal pain	26 (96.15%)
Fever	7 (26.9%)
Anorexia	5 (19.2%)
Nausea	12 (46.15%)
Vomit	7 (26.9%)
Diarrhoea	3 (11.5%)
Intestinal bleeding	1 (3.8%)

**Figure A1:**
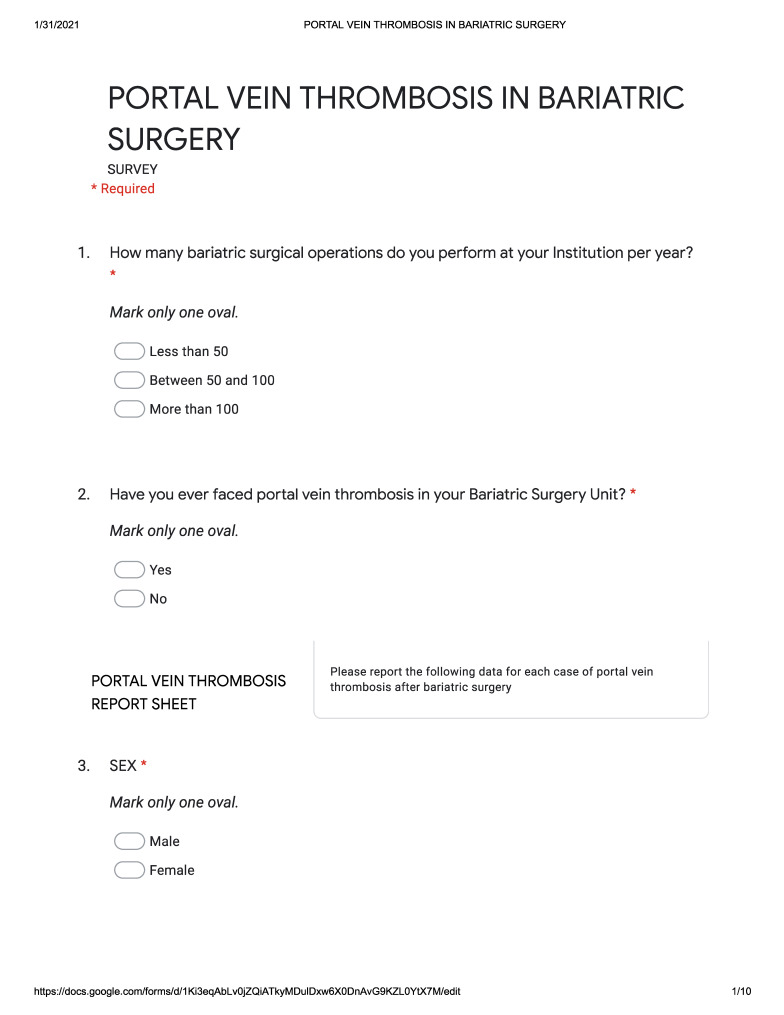

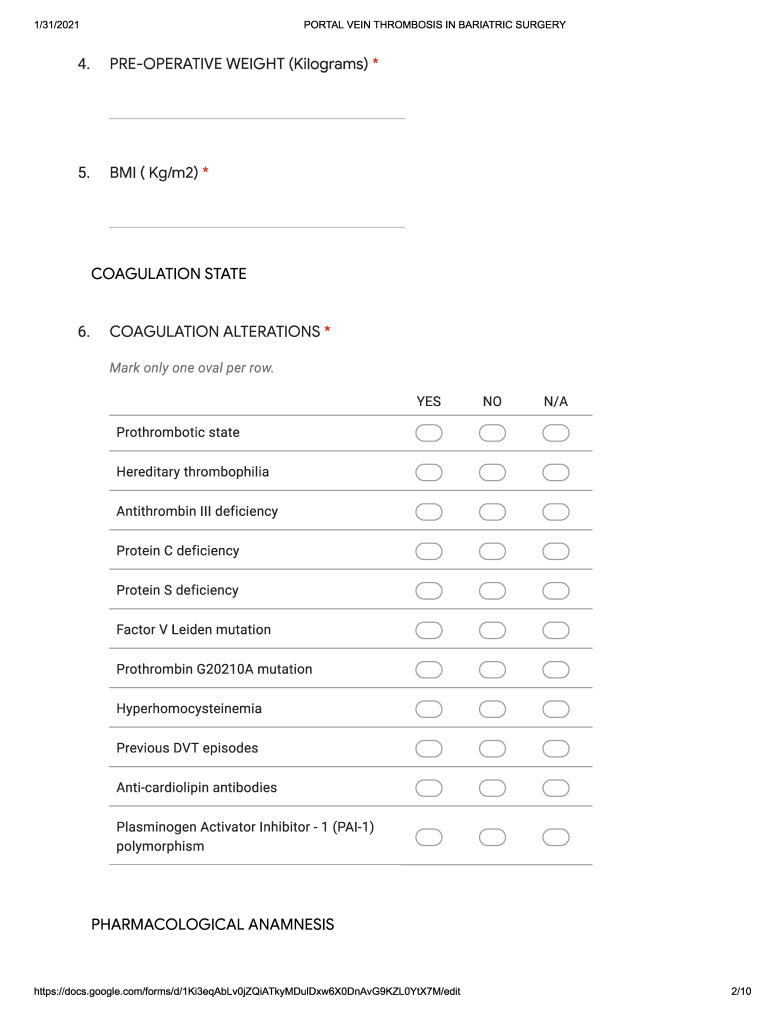

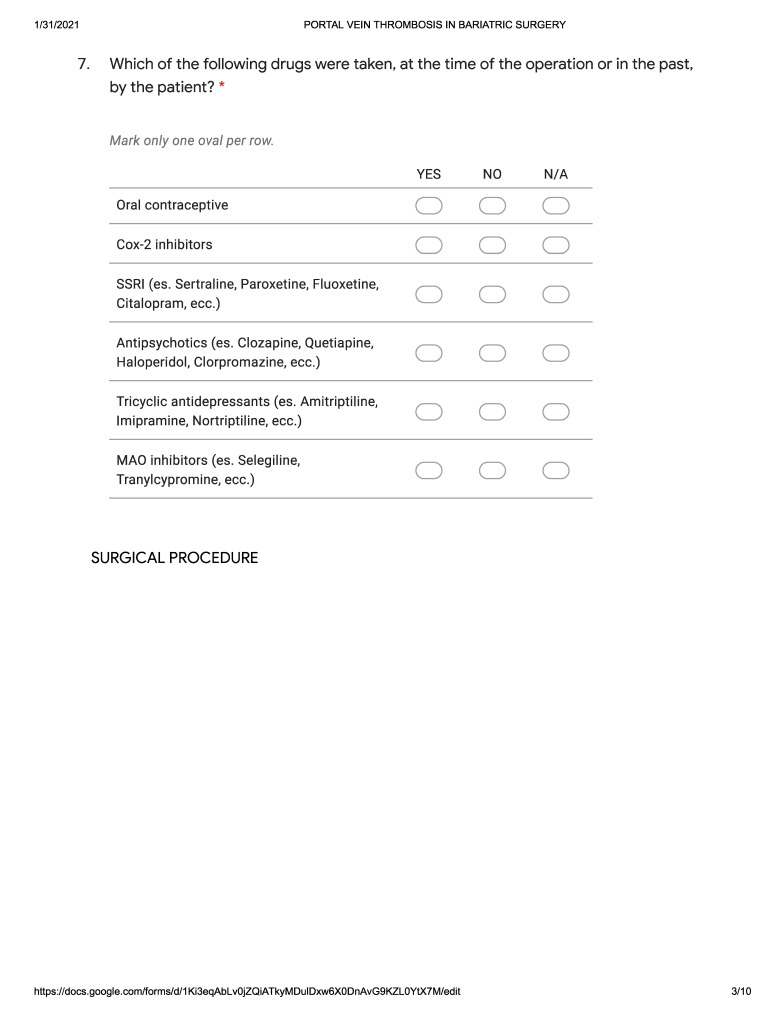

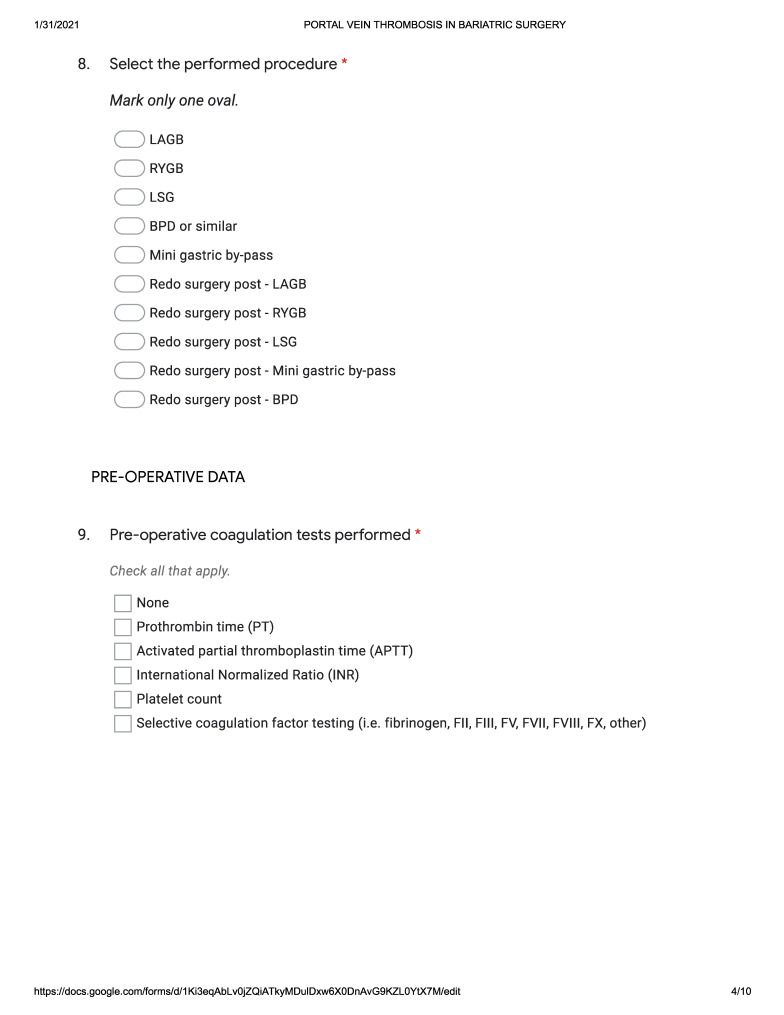

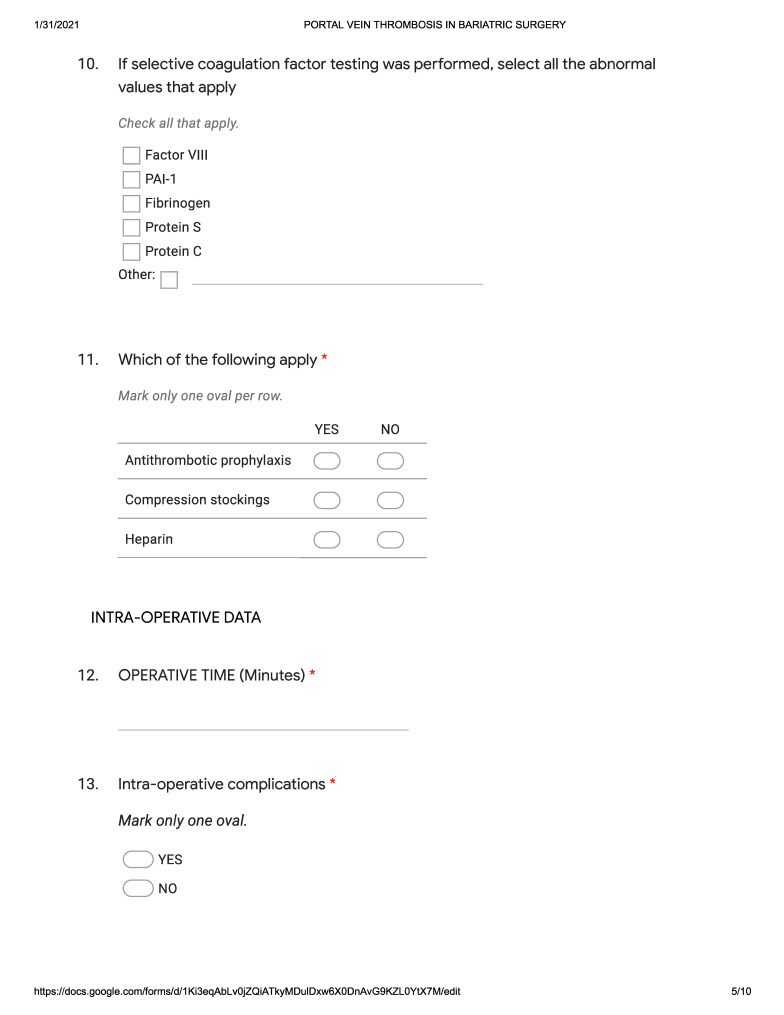

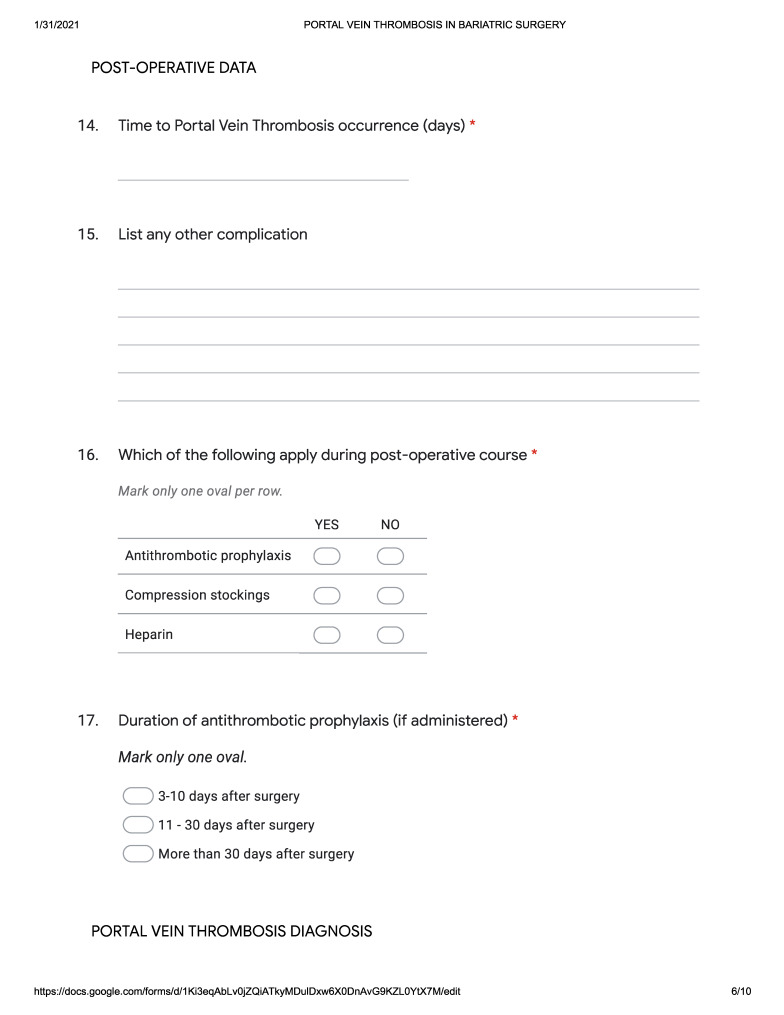

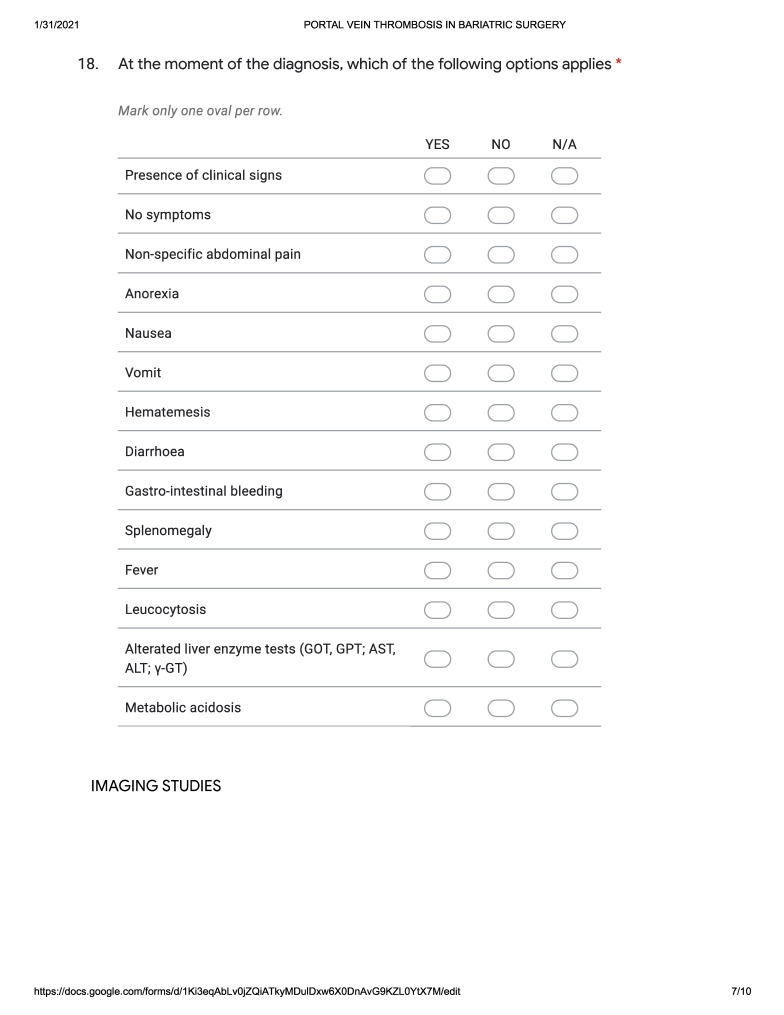

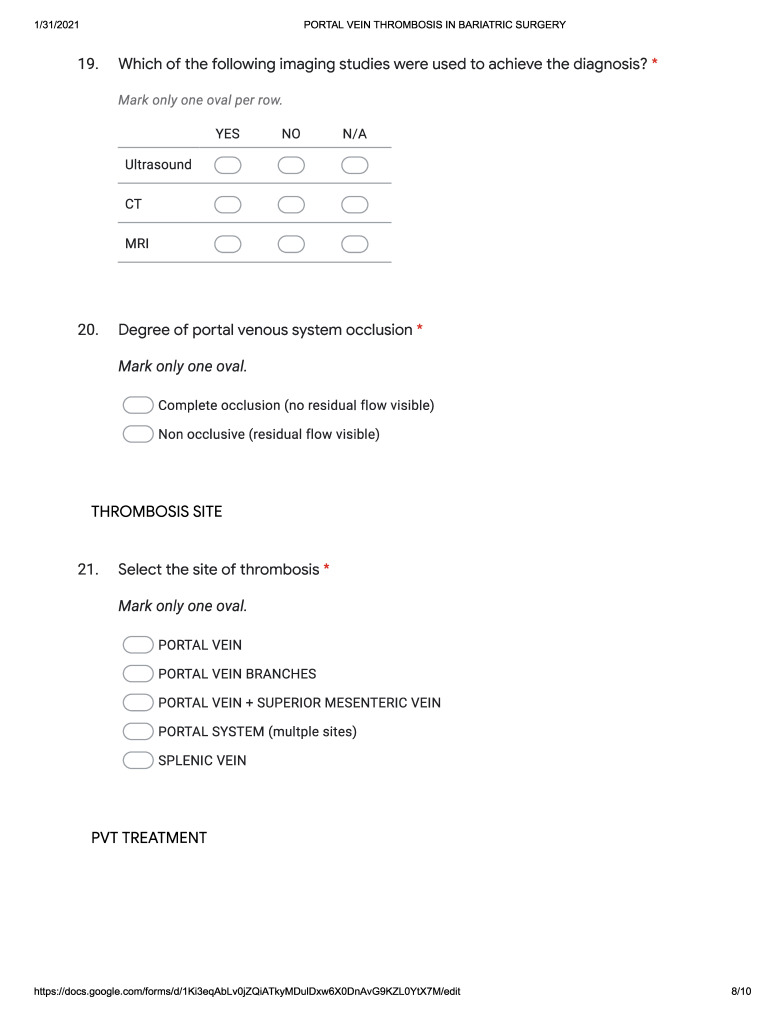

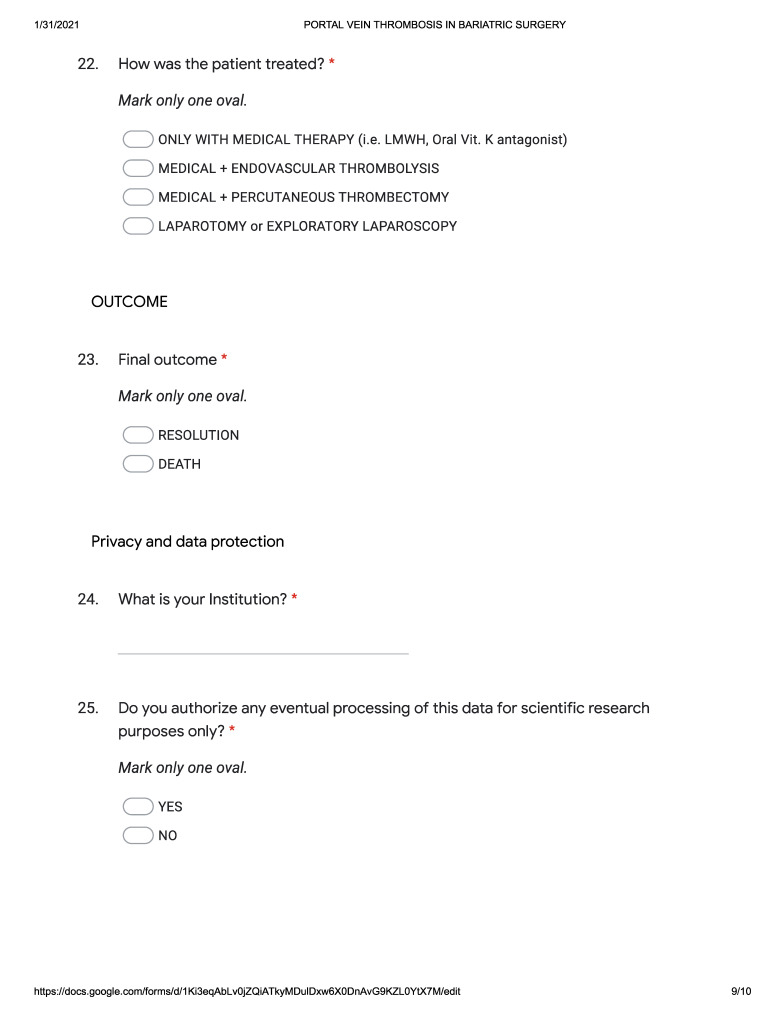

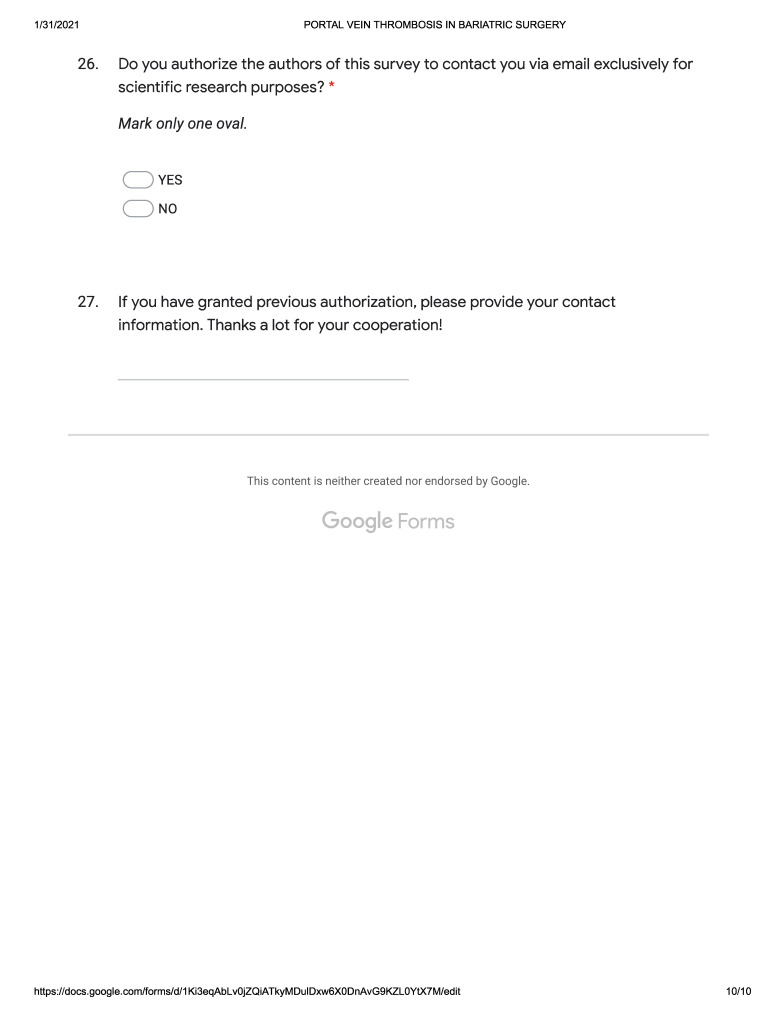
Portal vein thrombosis in bariatric surgery.

## Data Availability

The data presented in this study are available on request from the corresponding author. The data are not publicly available due to privacy restrictions.
